# Does increased lymphatic density contribute to fast drainage and metastatic spread to sentinel lymph nodes in melanoma?

**DOI:** 10.1038/sj.bjc.6601917

**Published:** 2004-06-08

**Authors:** D L Munz

**Affiliations:** 1 Clinic for Nuclear Medicine, Charité-University Medicine Berlin, Schumannstrasse 20/21, D-10117 Berlin, Germany

**Sir,**

The most common site of metastatic disease in melanoma is regional lymph nodes. Several well-documented factors predictive of regional nodal metastasis particularly include tumour ulceration and thickness (Balch *et al*, 2003). I read with great interest the article of Shields *et al* (2004), who have proven that lymphatic capillary density can be used to discriminate between melanomas that will subsequently metastasise and ones that will not. Their prognostic index based on immunohistochemically measured lymphatic capillary density in combination with lymphatic invasion and tumour thickness enables clear discrimination between metastatic and nonmetastatic human malignant melanoma. Lymphatic capillaries are the functional units of the lymphatic system and form the drainage pathways for interstitially injected radiocolloid in lymphoscintigraphy. Lymphoscintigraphy enables us to identify and localise the sentinel lymph nodes of primary tumours exhibiting lymphogenic metastatic potential. The findings of Shields *et al* (2004) now provide immunohistochemical evidence that helps explain the results of our recent prospective lymphoscintigraphic feasibility study on 276 melanoma patients (Maza *et al*, 2003). We found a relationship between the speed at which sentinel lymph nodes appear on dynamic lymphoscintigraphic scans and their metastatic involvement. The faster the lymphatic drainage takes place, the greater the metastatic involvement of the sentinel lymph nodes seems to be. The higher the lymphatic capillary density, the more the radiocolloid can presumably be absorbed. In other words, lymphatic capillary density may provide a histopathological explanation for the mechanisms involved in lymphoscintigraphic drainage speed. Hence, consistent evidence has been presented that lymphogenic pathway studies can yield predictors of metastatic spread. A fast scintigraphic appearance time, assessed under standardised conditions (Maza *et al*, 2003), seems to provide a new parameter for predicting the primary pathway and the likelihood of lymphogenic metastatic spread to allow surgeons and pathologists to better focus on determined lymph nodes. Now, it remains to be elucidated if sentinel lymph nodes can be excised in chronological order of their scintigraphic appearance, thereby possibly avoiding excision of slowly draining nodes. This approach would minimise morbidity and enhance diagnostic accuracy, particularly in multidirectional lymphatic drainage systems with sentinel lymph nodes in different basins.
